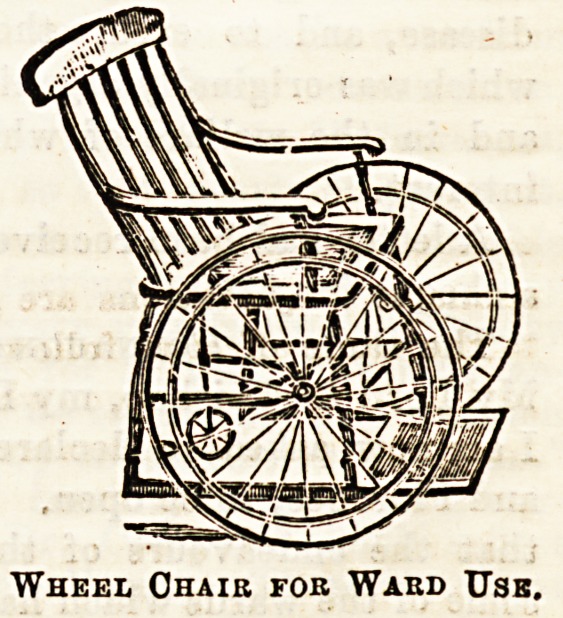# Practical Departments

**Published:** 1896-02-29

**Authors:** 


### PRACTICAL DEPARTMENTS.

### INVALID COUCH AND CHAIR.

Messrs. Farmer, Lane, and Co., of New Oxford Street, have always plenty of the newest in hospital and invalid furniture in stock. The couch illustrated below is made to combine most present-day improvements, and has been found very suitable for ward use, more than one having been supplied to the Middlesex Hospital, as well as to others in London and elsewhere. The particular advantage claimed for this couch is that it is fitted with swivel wheels for moving from place to place, the ordinary legs used when in a stationary position folding back, and being secured by a hook when the couch is to be put in motion. When the legs are lowered, which is done quite easily, the couch does not rest on the wheels at all, and is therefore quite firm and secure. Of course the back can be adjusted at any angle, and the foot-rest is removable at will. A book-rest, as shown in the drawing, can be fixed to the arm on either side. The wheels are rubber-

Couch with Swivel Wheels.

Wheel Chair for Ward Use.

tyred. The couch may be had upholstered in materials of varying quality, the price ranging in proportion from £12 12s. to £16 16s.

The wheel-chair illustrated in the second drawing is of a kind almost too well known to need commendation, but that every year sees some slight improvement which adds to its usefulness for ward work. These chairs are now so made as to be very light to move, and with sliding foot-rest, and rubber-tyred wheels, are comfortable, and can be easily propelled by the patient.

## Figures and Tables

**Figure f1:**
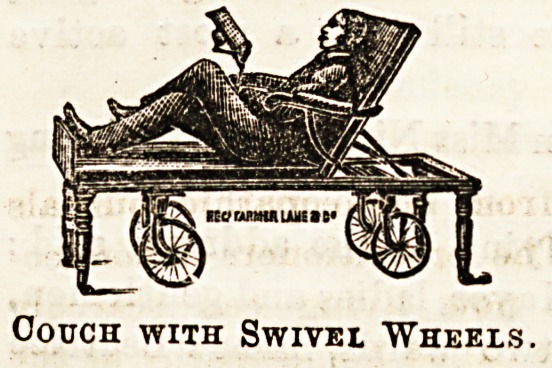


**Figure f2:**